# Extremely thin intermetallic layer in dissimilar AA6061-T6 and mild steel friction stir lap welding using a hemispherical tool

**DOI:** 10.1038/s41598-024-52412-w

**Published:** 2024-01-19

**Authors:** Danilo Ambrosio, Yoshiaki Morisada, Kohsaku Ushioda, Hidetoshi Fujii

**Affiliations:** Joining and Welding Research Institute, Osaka University, Ibaraki, Osaka 560-0047 Japan

**Keywords:** Engineering, Materials science

## Abstract

The dissimilar friction stir lap welding of AA6061-T6 and mild steel using the hemispherical tool tilted towards the retreating side is investigated. Critical defects such as hook features and internal voids are avoided by limiting the plunge depth in the lower plate to a tenth of a millimeter. The low heat generation guaranteed by the hemispherical tool produces a nanoscale intermetallic compound layer alternatively composed of an Al-rich and a ternary Al–Fe–Mg phases. The complex and extremely thin interlayers strengthen the Al–Fe mechanical bonding, guaranteeing high mechanical properties and rupture within the Al-stirred zone. Thermomechanical phenomena governing friction stir lap welding with the hemispherical tool drastically limit the growth of intermetallics, leading to the high mechanical strength of the lap joint.

## Introduction

The joining of dissimilar materials has increased significantly in recent decades^1^. Car manufacturers’ interest in reducing the maximum weight of vehicles is pushing the joining of steel and aluminum alloy blanks in different thicknesses, exploiting both the lower density of aluminum while taking advantage of good mechanical properties and the lower cost of steel^2^. Welding aluminum alloys and steel by fusion welding techniques presents several problems, such as different melting points, risk of porosity and cracking during solidification, formation of brittle intermetallic compound (IMC), and significant residual stress^3^. Such issues can be partially overcome through friction stir lap welding (FSLW). This solid-state welding solution can prevent problems related to the very high temperatures and the consequent melting of the base materials^4^. Under suitable welding parameters, the base materials can be welded without melting the base materials, achieving high-strength joints^5^. The main concerns for FSLW joint quality are voids at the interface, hook features, and IMC. The internal flaws reduce the joints’ strength^6^. The hook features must be either minimized^7^ or, if produced, ensure that they act as mechanical interlocking between the upper and lower plates^8^ instead of potential crack propagation points^9^. Instead, concerning IMC, the final impact on the Al–Fe lap joint is still debated. The formation of Fe or Al-rich IMC layers at the interface of the lap joints is strongly dependent on amount of heat generated during the process^10^. However, low temperatures might not guarantee the required softening of the base materials for correct stirring, leading to internal voids^11^. The Fe-rich intermetallics are more ductile in nature compared to the Al-rich intermetallics^12^ and consequently less dangerous for the lap joint. Some researchers reported that IMC layers are detrimental^13^, whereas others claimed that they are non-influential^14^ to the friction stir lap joint strength. Geng et al.^15^ proposed an optimal IMC layer thickness range between 1 and 2 $\upmu$m to maximize the mechanical strength of lap joints, reporting a drastic drop of load bear by the lap joint if thinner or no IMC was found at the interface. However, some researchers reported adequate mechanical bonding between Al and Fe blanks in Al–Fe lap welds based on solid-state welding techniques, even without IMC layers. Ogura et al.^16^ found an amorphous layer at the interface of the Al–Fe friction stir lap joint explained through mechanical alloying that occurs below the probe tip, formed right before the appearance of intermetallic compounds. In their experiments, the probe tip only rubbed the upper surface of the steel blank. A similar outcome was reported by Sun et al.^17^, reporting the occurrence of an amorphous layer at the interface during the flat spot friction stir welding of Al–Fe blanks. Even in this case, the tool did not plunge within the steel lower plate. The mechanical bond achieved during the spot welding was considerably high. Hence, both studies disprove other works claiming that a minimum IMC thickness was required to obtain good mechanical properties in Al–Fe lap welding. Hence, the role of IMCs on dissimilar Al/Fe lap joints’ strength still needs to be clarified. According to previous studies, very thick intermetallic layers are detrimental to the lap joints and are controlled by heat generation during welding. The hemispherical tool might limit heat input, strengthening the bonding between aluminum alloy and steel by avoiding thick, brittle IMC layers.

In the present study, lap welds are manufactured via friction stir welding using a hemispherical tool tilted towards the retreating side (RS). The feasibility of the hemispherical tool tilted towards RS for friction stir processing has been recently demonstrated^18^. To wholly exploit the new friction stir welding solution, it is here extended to dissimilar Al–Fe lap welding since the hemispherical tool shape might positively affect the formation of IMC layers and hook features. Additionally, from the manufacturing perspective, the new shape may be more attractive due to its flexibility compared to conventional FSW tools since it can successfully weld multiple blanks’ thicknesses using the same tool.

## Methods

**Figure 1:**
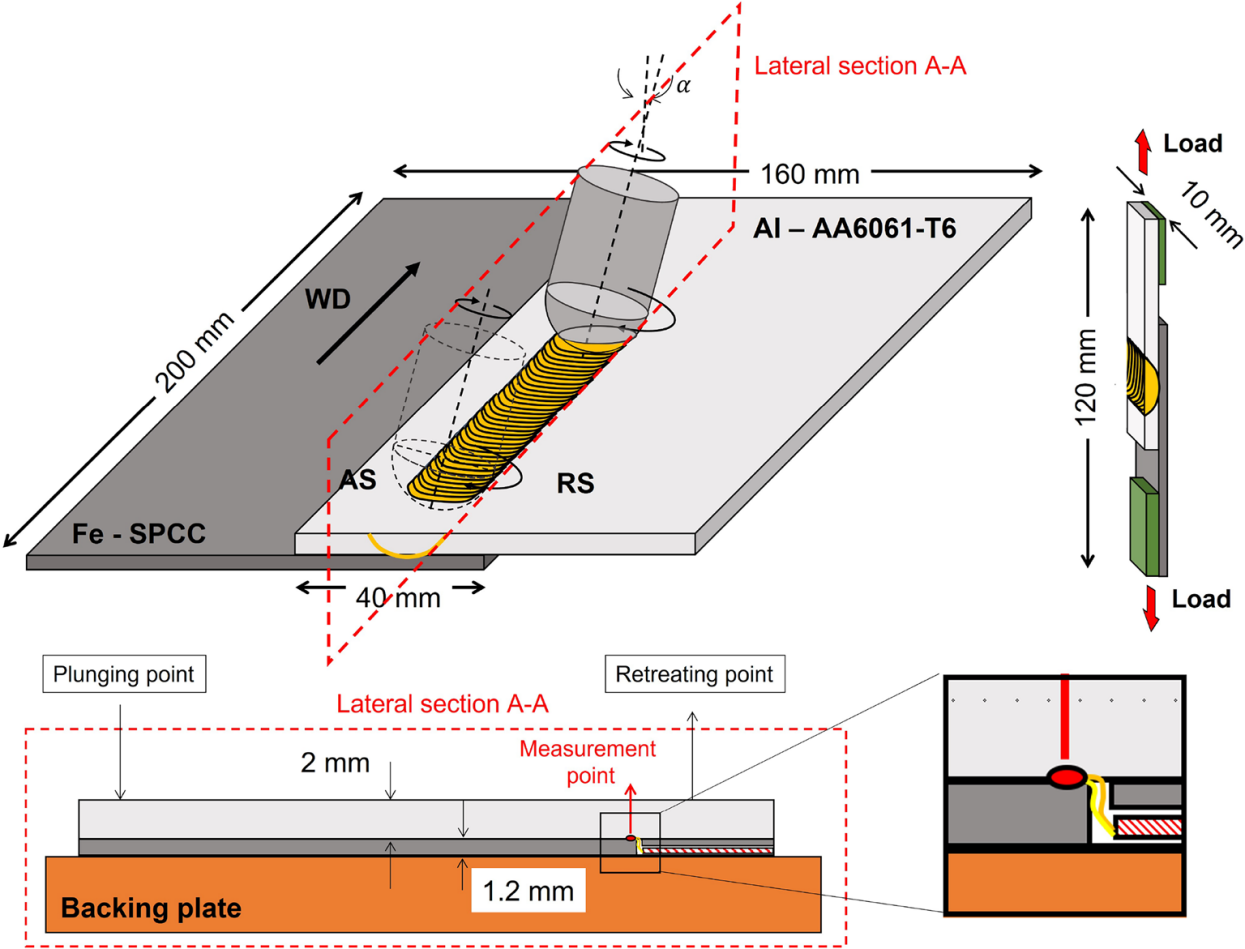
FSLW with hemispherical tool: experimental setup and tensile sample sketches.

An aluminum alloy (AA) 6061-T6 and SPCC steel sheets (200 mm width and 100 mm long) of 2 and 1.2 mm thickness, respectively, are used in this study. A 7.5 mm radius tungsten carbide hemispherical tool tilted of 3$^{\circ }$ towards RS is employed for lap welding. Figure 1 shows the welding setup sketch.

The welding is carried out in position control with a plunge depth of 2.1 mm to guarantee the hemisphere tip rubbing the steel sheet’s upper surface. Rotational and welding speeds are set at 500 rpm and 50 mm/min, respectively. Microstructural observations, energy-dispersive X-ray spectroscopy (EDS) and electron backscatter diffraction (EBSD) analysis are carried out with a JEM-7001FA field emission scanning electron microscope (FE-SEM) equipped with the TSL OIM system. Transmission electron microscopy (TEM)—EDS observations are performed with a JEM-2100F microscope to characterize the Al–Fe interface. Shear lap tests are carried out on an Instron machine with a 100 kN load cell and cross-head speed of 1 mm/min at room temperature. The dimensions of the tensile specimens are displayed in Fig. 1. The temperature at the interface is measured by spot welding the thermocouple tip on the steel upper surface along the welding line. A small 1.5 mm hole is drilled in the steel plate, and the thermocouple tip wires pass through it and are welded to the steel plate to ensure its position along the welding line (Fig. 1).

## Results

**Figure 2:**
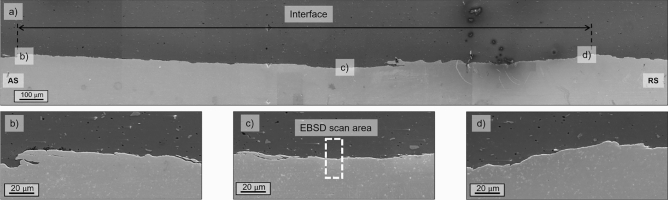
Lap joint: (**a**) cross-section interface and details (**b**) AS, (**c**) center, (**d**) RS.

Figure 2a–d shows the joint cross-section and interface details. The Al and Fe plate interface is uniform, continuous, and void-free. Few steel fragments are found within the upper Al matrix, revealing that the tool tip rubbed the lower steel plate without stirring. The limited plunge depth and the curved tool tip shape avoided pronounced hook features. Hence, the welding configuration hinders the two primary sources of premature failure in friction stir lap joints, such as voids and hook features. Both are avoided thanks to the selected plunge depth, which avoids the plunging and stirring of the steel lower plate. The former impedes the significant penetration of the tool in the lower plate with the consequent upward bending of the sheet interface favouring steel hook within the Al upper plate^19^. The latter avoids generating steel fragments in the Al stirred zone, which have been proven as a source of micro-voids in friction stir lap welds^20^.

**Figure 3:**
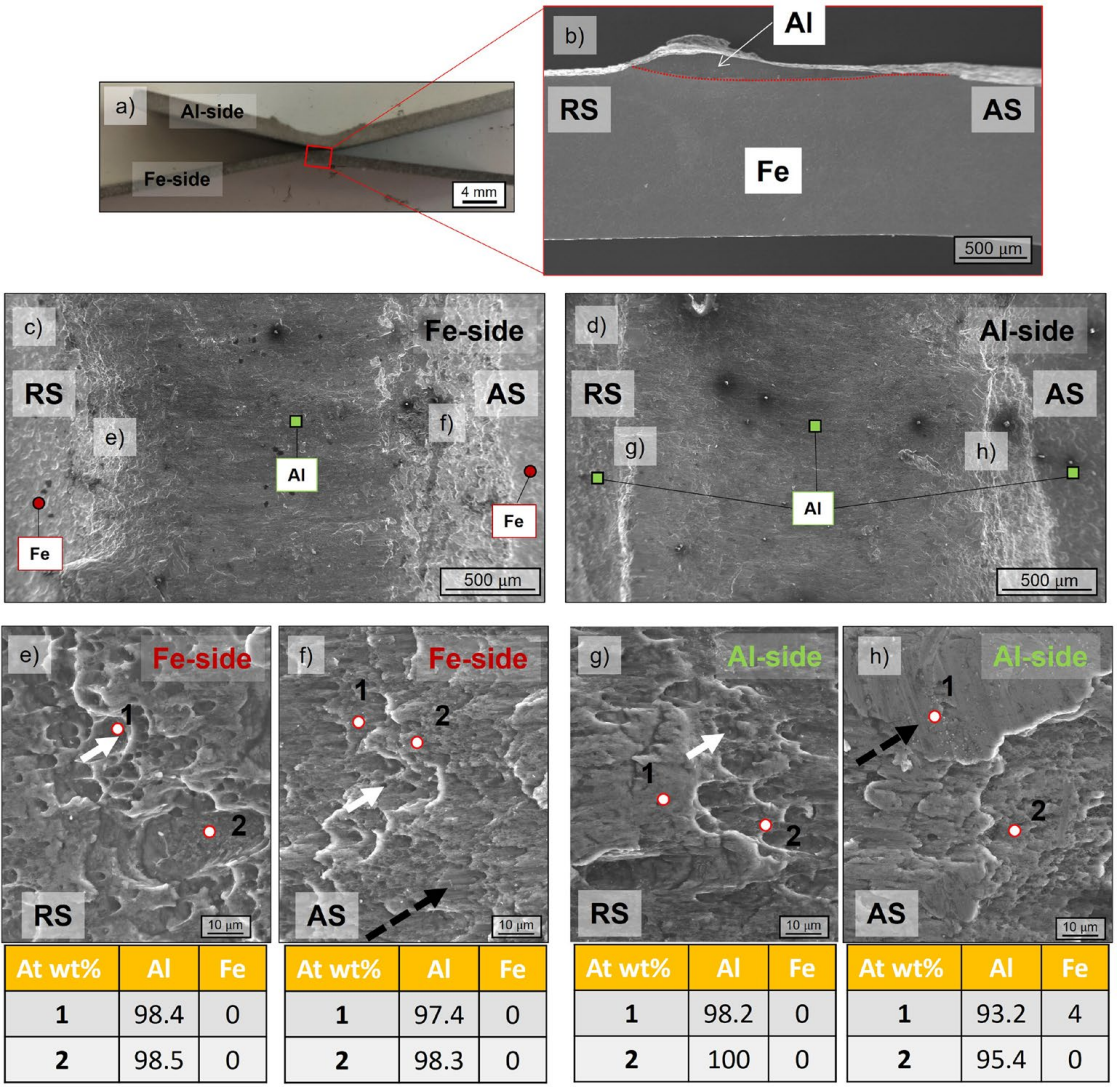
Details of the fractured surfaces: (**a**) macroview of the fractured sample, (**b**) detail of the lower part of the fractured sample, (**c**) lower fractured surface, (**d**) upper fractured surface, (**e**) RS detail Fe side, (**f**) AS detail Fe side, (**g**) RS detail Al side, (**h**) AS detail Al side.

The three-lap shear tests result in an average joint efficiency (calculated according to^8^) of 70%, which aligns with the highest strength in the literature (between 70 and 80%) for conventional friction stir lap welding. The high strength agrees with the interface details observed, such as the absence of both gaps at the interface and hook features at RS and AS, ensuring an efficient mechanical bonding without any potential stress concentration point. Figure 3a displays the fractured sample.

The fracture occurs within the stirred zone (SZ) of the Al plate rather than the interface, as highlighted in Fig. 3b, confirming the strong mechanical bonding between the two plates. EDS point scans are performed in the areas highlighted in Fig. 3c,d. The EDS point scans revealed the high atomic percentage in weight Al and are reported in Fig. 3e–h. They confirm the fracture occurred completely in the Al rather than interface or eventual IMC layers. Dimples (solid white arrows) and cleavage facets (dotted black arrows) can be observed in the details in Fig. 3e–h. At RS (Fig. 3e,g), there is a higher concentration of dimples, whereas at the center and AS (Fig. 2f,h), more cleavage facets appear. According to both features, the fracture can be considered ductile-brittle, with void generation and growth beginning at RS and then the rapid propagation within the SZ promoting brittle fracture and consequent cleavage facets.

**Figure 4:**
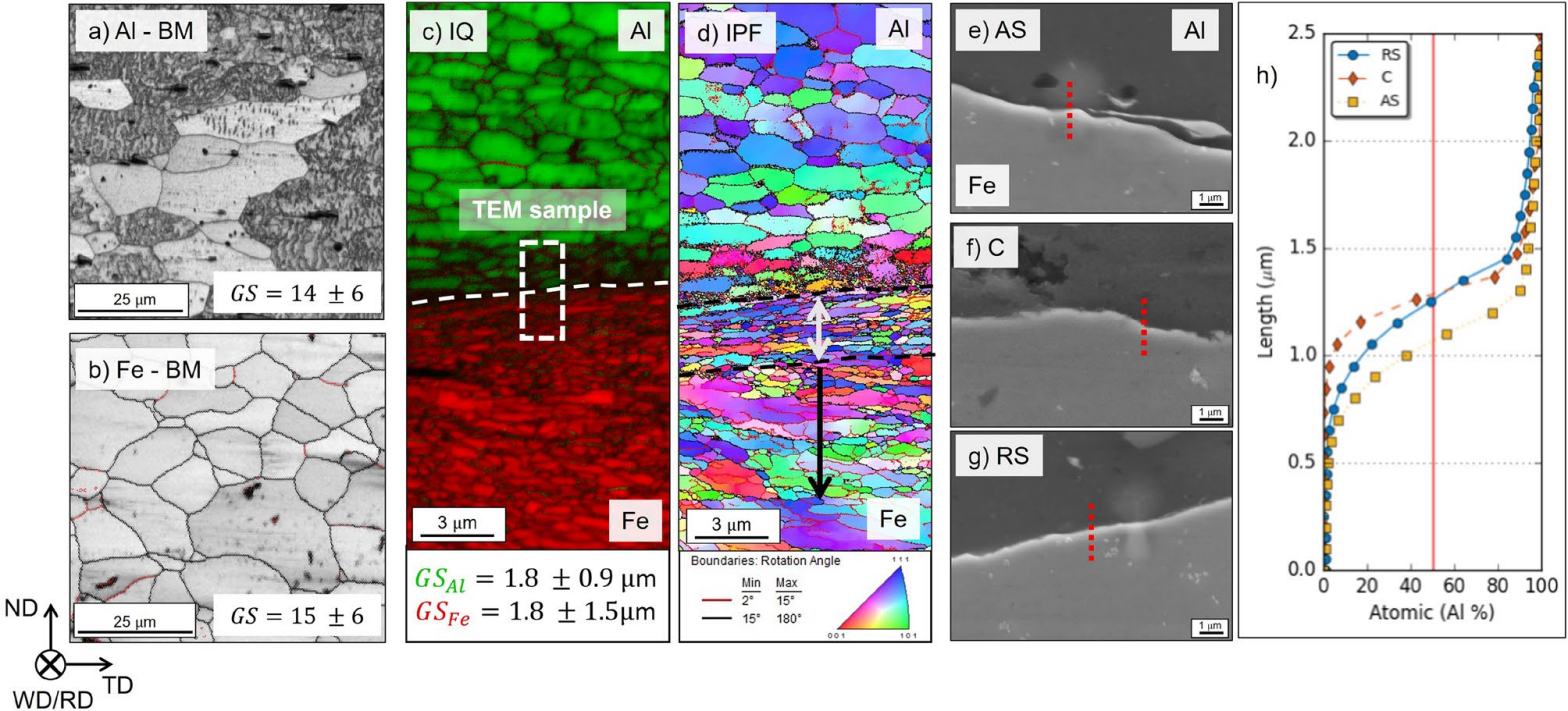
Image quality from EBSD scans of (**a**) Al base material, (**b**) Fe base material, (**c**) interface and (**d**) inverse pole figure (IPF) interface. SEM images highlighting the EDS line scans at (**e**) AS, (**f**) C, (**g**) RS and (**h**) Al atomic concentration along the lines.

The microstructural modifications induced by the hemispherical tool stirring the aluminum and rubbing the steel surface are discerned by comparing base material Al and Fe microstructures (Fig. 4a,b) with the interface of the lap joint (Fig. 4c,d). EBSD scan is carried out at the center of the joint interface and displayed in Fig. 2c.

The process significantly affects the aluminium microstructure, presenting the typical features of grains in the SZ of friction stir weld, such as equiaxed and refined grains with a drastic reduction in grain size due to dynamic recrystallization, as highlighted in Fig. 4c. Focusing on the steel, the gradient in grain size can be perceived with grains significantly small near the interface while increasing their size by moving away from it. Hence, the impact of the process on the steel microstructure is limited to a few microns. At the interface, the microstructure is affected by the forging action of the hemisphere and the high temperature, producing a recrystallized microstructure with small grains characterized by high-angle boundaries (grey double arrow in Fig. 4d), while after a few micrometers, grains appear partially recrystallized elongated orthogonally to the compression direction with a high fraction of low angle boundaries between 2 and 15$^{\circ }$ (area highlighted by the black arrow in Fig. 4d). These features confirm that the chosen plunge depth and the shape of the hemispherical tool avoided steel stirring, with the tool tip only rubbing and forging the lower steel plate. Additionally, SEM observations and EDS line scans are performed at the advancing side (AS), center (C), and retreating side (RS) of the interface to reveal the chemical composition in atomic percent at the interface. SEM images are shown in Fig. 4e–g for AS, C and RS, respectively. The net transition between Al and Fe is observed in each figure, and any IMC layers can be identified. The red dotted lines represent the EDS line scans of 25 points over a 2.5 $\upmu$m distance. Figure 4h displays the evolution of Al atomic percentage along the three lines (the counterpart is Fe and a very small percentage of Mg). As expected, Al atomic concentration decreases in the diffusion layer from the upper to the lower plate, with a gradual transition given by the solid diffusion occurring at the interface. However, small plateaus of Al concentration generally corresponding to IMC layers^14^ are not detected. Hence, the EDS line scans corroborate the information withdrawn from SEM images.

**Figure 5:**
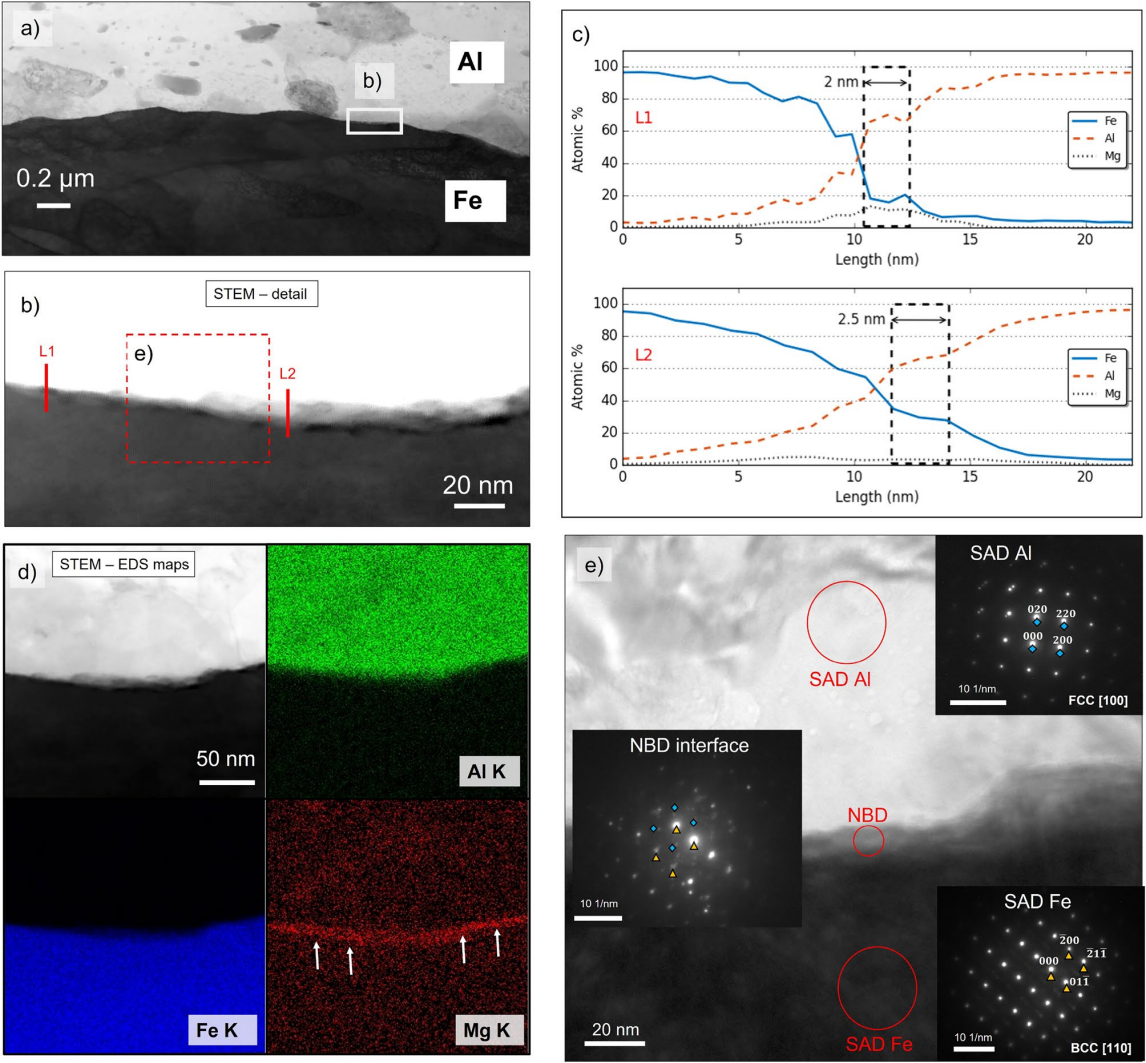
TEM analysis: (**a**) STEM low magnification observation of the interface, (**b**) local detail highlighted in (a) at two different contrast/brightness levels to highlight the interlayer, (**c**) chemical composition in atomic percent along L1 and L2 and (**d**) EDS map of (b), (**e**) HR-TEM photograph of the interface and corresponding selected area electron diffraction (SAD) and nano-beam diffraction (NBD) patterns of Al, Fe, and interface).

The bonding between Al and Fe is further analyzed through TEM observations. The sample is taken at the center of the joint interface and highlighted in the EBSD scan in Fig. 4c. Figure 5a displays the interface detail taken at the center of the bonded area. The transition between aluminum and steel is sharp at this magnification, and no layer is observed. Higher magnification of the spot highlighted with the solid white rectangle in Fig. 5a is shown in Fig. 5b. The gray-shaded discontinuous interlayer between Al and Fe is worth noting. Its maximum thickness measured is 9 nm. Two EDS line measurements are carried out to study the chemical composition at the interface. One crosses the interface at the sharp Al/Fe transition (L1), while the other passes through the gray-shaded interlayer (L2). Twenty-five points are taken over a length of 22 nm. The chemical composition (in atomic percent) evolution through the length for both L1 and L2 of Al, Fe and Mg are displayed in Fig. 5c.

The atomic percentage of Al and Fe evolve similarly in both cases, and it is worth noting that, within the Al side, an extremely thin IMC layer is detected, which extends for 2 and 2.5 nm for L1 and L2, respectively. Hence, according to the EDS measurements, the interlayer highlighted in Fig. 5b cannot be considered an IMC layer. However, extremely thin nanoscale IMCs at the interface form and are highlighted in the curves in Fig. 5c with the dashed black rectangles. According to the different chemical compositions, different phases formed at the interface. In the case of L1, it is worth noting the peak in Mg atomic concentration, which reaches 13% in the same zone in which the IMC is detected, revealing a local high concentration of Mg at the interface. The EDS map scan in Fig. 5d confirms the local high concentration of Mg at the interface (as highlighted by the solid white arrows). Figure 5e shows the high-resolution (HR) STEM micrograph of the area highlighted by the dashed red rectangle in Fig. 4b. Steel and aluminum can be distinguished with an interlayer in the transition zone. Their electron diffraction patterns displayed in Fig. 5e are typical of face-centered cubic (FCC) (along <100>^21^) and body-centered cubic (BCC) (along <110>^22^) lattices, respectively. The diffraction pattern in the interlayer is typical of crystalline structure, precluding the hypothesis of an amorphous layer, as reported in other cases of overlap Al–Fe welds^16,17^. However, identifying a specific lattice structure to correlate with a phase is challenging since the nano-beam diffraction (NBD) pattern comprises superimposed patterns as indicated by blue and yellow spots highlighted in Fig. 5e.

## Discussion

**Figure 6:**
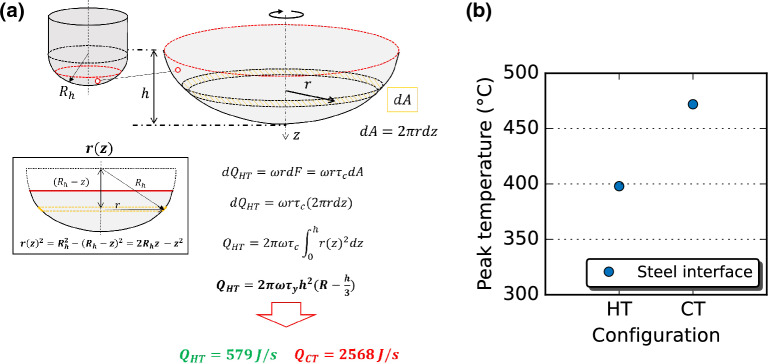
Heat generation: (**a**) estimation showing the estimated interaction area and the analytical formulation of heat generation for the hemispherical tool, (**b**) temperatures measured at the Al/Fe interface.

It is worth noting that the modified version of friction stir lap welding using a hemispherical tool tilted towards RS, with the tool tip rubbing the steel surface, leads to a sound lap joint. Joint efficiency reaches 70%, with the fracture occurring within the Al stirred zone. The benefits of the new tool shape and geometry hinder rupture at the interface. The low plunge depth and the shape of the tool limit hook features. Internal voids are prevented due to the lack of Fe stirring. Extremely thin, continuous, and intermittent IMC layers around 2 nm are found through TEM observations. The Al-rich IMC phase along scan line L2 agrees with previous studies on dissimilar Al/Fe FSLW. According to the atomic weight concentration range and prior works, it can be speculated that the phase is Al$_5$Fe$_2$^23^. Instead, the ternary Al–Fe–Mg phase revealed by the L1 line scan has never been reported before to the authors’ knowledge in dissimilar AA6061-T6 and mild steel friction stir welding. However, local Mg, Cu, and Zn enrichment have already been reported in dissimilar friction stir^24^ and linear friction^25^ welding of AA7075 and mild steel. The minimal formation of IMC layers might be related to the low heat generation given by the hemispherical tool. The heat generated by the hemispherical tool (HT) can be estimated using the same analytical approach proposed by Schmidt et al.^26^ for conventional tools (CT). The tool portion interacting with the workpiece (below the red dashed circle) and the heat generation ($Q_{HT}$) calculation is displayed in Fig. 6.

The hypotheses for the heat generation calculation are as follows:

- sticking condition, i.e., shear stress at the contact ($\tau _c$) equal to the shear yield strength ($\tau _y$) of the material;
- $\tau _y$ depends on the stirred material (AA6061) temperature, which has been approximated to 400 $^{\circ }$C (based on measurement at similar welding conditions^27^), and the flow stress at 400 $^{\circ }$C is taken from^28^;
- tool tip completely in contact with aluminum, only rubbing the steel upper surface, with a plunge depth of 2.1 mm.

With these hypotheses, the heat generation has been estimated for the hemispherical tool, neglecting for simplicity the tilting of 3$^{\circ }$ towards RS, and for a conventional tool geometry used for lap welding of the same thicknesses and similar base material (2 mm and 1.2 mm for, Al and Fe plates, respectively) found in the literature (shoulder diameter of 15 mm, probe diameter and height of 6 mm and 2.2 mm, respectively)^29^. In the case of the hemispherical tool, heat generation is equal to 579 J/s against 2568 J/s of the conventional tool. Temperature measurements at the interface along the welding line have been performed during welding with the hemispherical and conventional tools, with conventional tool dimensions according to the ones used for the analytical estimation of heat input^29^. The values recorded are 398 and 472 $^{\circ }$C for the hemispherical and conventional tools, respectively, and reported in Fig. 6. Hence, as hypothesized, the selected hemispherical geometry can guarantee the correct material flow to fill the cavity left by the tool while advancing, avoid internal voids at the interface, and generate less heat due to a lower tool-workpiece interaction surface. Consequently, welding with a 7.5 mm radius hemispherical tool in the selected welding parameters leads to a nanoscale intermetallic layer at the interface, strengthening the mechanical bonding between Al and Fe.

## Conclusion

The hemispherical tool can be considered a viable solution for dissimilar AA6061 and mild steel lap welding. The thermomechanical conditions developed during the welding in the selected welding parameters led to a strongly bonded interface characterized by an extremely thin IMC layer (between 2 and 2.5 nm), minimal hook features, and no internal voids either at the interface or in the Al stirred zone, with an average joint efficiency of 70%. This result is unusual, considering that Al–Fe lap joint strength has often been claimed to drop significantly when IMC thickness is below the micron scale. The continuous IMC layer is composed alternatively of an Al-rich phase, and a ternary Al–Fe–Mg phase that might be responsible for strengthening the interface bonding, leading to the high mechanical properties and the fracture within SZ. The limited IMC generated with the hemispherical tool is given by the very low heat generation guaranteed by the lower tool surface interacting with the workpiece, leading to a significant drop in peak temperature from 472 $^{\circ }$C for conventional to 398 $^{\circ }$C for the hemispherical tool. Hence, using a hemispherical tool tilted towards RS, the low heat generation reduces the maximum temperature reached at the interface, limiting the growth of the intermetallic compound layer. The nanoscale IMC strengthens the Al/Fe bond, guaranteeing a lap joint’s efficiency of 70% and fracture within the Al stirred zone. Further research is necessary to clarify the bonding mechanism produced with the hemispherical tool, aiming at even higher bonding efficiency by varying welding parameters and modifying the hemisphere radius.

## Data Availability

The datasets used and/or analysed during the current study available from the corresponding author on reasonable request.

## References

[1] Zhang G, Su W, Zhang J, Wei Z (2011). Friction stir brazing: A novel process for fabricating Al/steel layered composite and for dissimilar joining of Al to steel. Metall. Mater. Trans. A Phys. Metall. Mater. Sci..

[2] Taban E, Gould JE, Lippold JC (2010). Dissimilar friction welding of 6061-T6 aluminum and AISI 1018 steel: Properties and microstructural characterization. Mater. Des..

[3] Maurya AK, Pandey C, Chhibber R (2021). Dissimilar welding of duplex stainless steel with Ni alloys: A review. Int. J. Press. Vessels Pip..

[4] Huang Y (2018). Probe shape design for eliminating the defects of friction stir lap welded dissimilar materials. J. Manuf. Process..

[5] Thomas WM, Staines DG, Norris IM, de Frias R (2003). Friction stir welding tools and developments. Weld. World.

[6] Simar A, Avettand-Fènoël MN (2017). State of the art about dissimilar metal friction stir welding. Sci. Technol. Weld. Join..

[7] Dubourg L, Merati A, Jahazi M (2010). Process optimisation and mechanical properties of friction stir lap welds of 7075-T6 stringers on 2024-T3 skin. Mater. Des..

[8] Wang T (2018). Friction stir scribe welding technique for dissimilar joining of aluminium and galvanised steel. Sci. Technol. Weld. Join..

[9] Geyer M, Vidal V, Pottier T, Boher C, Rézaï-Aria F (2021). Investigations on the material flow and the role of the resulting hooks on the mechanical behaviour of dissimilar friction stir welded Al2024-T3 to Ti–6Al–4V overlap joints. J. Mater. Process. Technol..

[10] Kimapong K, Watanabe T (2005). Lap joint of A5083 aluminum alloy and SS400 steel by friction stir welding. Mater. Trans..

[11] Ambrosio D, Morisada Y, Ushioda K, Fujii H (2023). Material flow in friction stir welding: A review. J. Mater. Process. Technol..

[12] Wang Y, Zhou S, Vecchio KS (2016). Annealing effects on the microstructure and properties of an Fe-based metallic-intermetallic laminate (MIL) composite. Mater. Sci. Eng., A.

[13] Kimapong K, Watanabe T (2005). Effect of welding process parameters on mechanical property of FSW lap joint between aluminum alloy and steel. Mater. Trans..

[14] Pourali M, Abdollah-zadeh A, Saeid T, Kargar F (2017). Influence of welding parameters on intermetallic compounds formation in dissimilar steel/aluminum friction stir welds. J. Alloys Compd..

[15] Geng P (2022). Elucidation of intermetallic compounds and mechanical properties of dissimilar friction stir lap welded 5052 Al alloy and DP590 steel. J. Alloys Compd..

[16] Ogura T (2012). Partitioning evaluation of mechanical properties and the interfacial microstructure in a friction stir welded aluminum alloy/stainless steel lap joint. Scr. Mater..

[17] Sun YF, Fujii H, Takaki N, Okitsu Y (2013). Microstructure and mechanical properties of dissimilar al alloy/steel joints prepared by a flat spot friction stir welding technique. Mater. Des..

[18] Ambrosio D, Sharma A, Mukuda M, Morisada Y, Fujii H (2024). Feasibility of friction stir welding using a hemispherical tool tilted towards the retreating side. J. Adv. Join. Process..

[19] Badarinarayan H, Shi Y, Li X, Okamoto K (2009). Effect of tool geometry on hook formation and static strength of friction stir spot welded aluminum 5754-O sheets. Int. J. Mach. Tools Manuf.

[20] Mahto RP, Kumar R, Pal SK (2020). Characterizations of weld defects, intermetallic compounds and mechanical properties of friction stir lap welded dissimilar alloys. Mater. Charact..

[21] Rack HJ, Krenzer RW (1977). Thermomechanical treatment of high purity 6061 aluminum. Metall. Trans. A.

[22] Liu X (2018). In situ heating TEM observations on carbide formation and alpha-Fe recrystallization in twinned martensite. Sci. Rep..

[23] Springer H (2011). On the formation and growth of intermetallic phases during interdiffusion between low-carbon steel and aluminum alloys. Acta Mater..

[24] Saleh M, Morisada Y, Ushioda K, Fujii H (2023). Improvement of weldability and fracture behavior of mild steel and A7075 aluminum alloy dissimilar friction stir welded joints by increasing welding speed. Mater. Today Commun..

[25] Khan F (2023). Sound dissimilar linear friction welding of A7075-T6 Al and mild steel by simultaneous interfacial deformation using higher forging speed. J. Manuf. Process..

[26] Schmidt H, Hattel J, Wert J (2004). An analytical model for the heat generation in friction stir welding. Model. Simul. Mater. Sci. Eng..

[27] Ambrosio D (2022). A semi-empirical model for peak temperature estimation in friction stir welding of aluminium alloys. Sci. Technol. Weld. Join..

[28] Ding S, Shi Q, Chen G (2021). Flow stress of 6061 aluminum alloy at typical temperatures during friction stir welding based on hot compression tests. Metals.

[29] Geng P (2022). Measurement and simulation of thermal-induced residual stresses within friction stir lapped Al/steel plate. J. Mater. Process. Technol..

